# Integrating necroptosis into pan-cancer immunotherapy: a new era of personalized treatment

**DOI:** 10.3389/fimmu.2024.1510079

**Published:** 2024-12-09

**Authors:** Yan Gao, Sheng Chen, Lei Li

**Affiliations:** ^1^ Department of Respiratory and Critical Care Medicine, The Affiliated Huai’an Hospital of Xuzhou Medical University, Huai’an, China; ^2^ Department of Thoracic Surgery, The Affiliated Huaian No.1 People’s Hospital of Nanjing Medical University, Huai’an, China

**Keywords:** necroptosis, machine learning, pan-cancer analysis, immune microenvironment, immunotherapy

## Abstract

**Introduction:**

Necroptosis has emerged as a promising biomarker for predicting immunotherapy responses across various cancer types. Its role in modulating immune activation and therapeutic outcomes offers potential for precision oncology.

**Methods:**

A comprehensive pan-cancer analysis was performed using bulk RNA sequencing data to develop a necroptosis-related gene signature, termed Necroptosis.Sig. Multi-omics approaches were employed to identify critical pathways and key regulators of necroptosis, including HMGB1. Functional validation experiments were conducted in A549 lung cancer cells to evaluate the effects of HMGB1 knockdown on tumor proliferation and malignancy.

**Results:**

The Necroptosis.Sig gene signature effectively predicted responses to immune checkpoint inhibitors (ICIs). Multi-omics analyses highlighted HMGB1 as a key modulator of necroptosis, with potential to enhance immune activation and therapeutic efficacy. Functional experiments demonstrated that HMGB1 knockdown significantly suppressed tumor proliferation and malignancy, reinforcing the therapeutic potential of targeting necroptosis.

**Discussion:**

These findings underscore the utility of necroptosis as a biomarker to guide personalized immunotherapy strategies. By advancing precision oncology, necroptosis provides a novel avenue for improving cancer treatment outcomes.

## Introduction

Immunotherapy has now become a cornerstone of modern oncology, fundamentally transforming the treatment of various malignancies (1, 2). Unlike traditional cancer treatments such as chemotherapy and radiation, immunotherapy harnesses the patient’s own immune system to fight tumors, making it not only more precise but also significantly less toxic (3, 4). In recent years, the rapid advancement of immunotherapy has not only provided substantial extensions in survival for many cancer patients but has also greatly improved their quality of life by reducing the side effects associated with conventional therapies (5, 6). Innovative forms of immunotherapy, including immune checkpoint inhibitors, CAR T-cell therapy, and cancer vaccines, have demonstrated groundbreaking results in clinical settings, particularly in some treatment-resistant cancers (7–9).

However, despite the impressive benefits of immunotherapy, the reality is that not all patients derive the same level of benefit (10, 11). In fact, only a subset of patients showing significant responses to these treatments, while many others exhibit limited or no response. The wide variability in patient responses to immunotherapy highlights the complexity and challenges of cancer treatment. This heterogeneity is influenced by various factors, such as genetic differences and variations in the immune microenvironment between individuals (12). Therefore, the identification and exploration of relevant biomarkers to accurately predict which patients are most likely to benefit from immunotherapy has emerged as a crucial area of ongoing research (13–16). By gaining deeper insights into these biomarkers, physicians can better tailor treatment plans to individual patients, ultimately improving the efficacy of immunotherapy and addressing issues such as immune resistance (17). Through this approach, the future of immunotherapy promises to be more precise, more effective, and capable of benefiting an even greater number of cancer patients (18).

Necroptosis is a form of programmed cell death similar to necrosis. When a cell fails to undergo apoptosis properly due to inflammation, oxidative stress, or ischemic stress, necroptosis is activated as an “alternative” to apoptosis (19). Unlike apoptosis, necroptosis does not rely on caspase activity but requires MLKL phosphorylation, regulated by RIPK3 (20, 21). This phosphorylation event causes MLKL to form pore complexes on the plasma membrane, leading to the release of DAMPs (damage-associated molecular patterns), cell swelling, and membrane rupture (22, 23). Most studies on the molecular mechanisms of necroptosis involve the tumor necrosis factor (TNF) signaling pathway. Typically, TNF induces an inflammatory response by activating pro-inflammatory genes through NF-κB signaling (24). Necroptosis is also triggered by death receptors on the cell membrane (such as TNFR1, DR4/5, and FAS receptors) and can be initiated by pattern-recognition receptors (PRRs). Downstream, necroptosis is regulated by three key molecules: MLKL (mixed lineage kinase domain-like pseudokinase), RIPK1 (receptor-interacting serine/threonine kinase 1), and RIPK3. These molecules can serve as potential biomarkers (21).

Necroptosis, unlike apoptosis, generates secondary messengers that interact with immune cells in the tumor microenvironment, signaling potential danger (25). This lytic form of cell death enhances the delivery of antigens and adjuvants to immune cells, potentially improving cancer therapies by integrating mechanisms of programmed cell death and immune activation. The findings indicate that necroptosis and its associated features may serve as valuable predictive biomarkers, with possible implications for other cancer types. Building on this discovery, we initiated a comprehensive project that integrates pan-cancer sample cohorts and bulk RNA sequencing datasets to explore, for the first time, the role of necroptosis in personalized cancer therapies, driven by its distinct molecular markers. By leveraging these two robust data sources, we aim to evaluate the clinical relevance of necroptosis across diverse cancers and gain deeper insights into its molecular pathways through multi-omics analyses. This integrated strategy seeks to establish a solid foundation for more precise, personalized cancer treatments, ultimately contributing to a refined framework for individualized cancer care. Finally, we validated through both *in vitro* and *in vivo* experiments that knocking down the HMGB1 gene, one of the modeling genes of Necroptosis.Sig, in the A549 lung cancer cell line can suppress the malignant biological behavior of tumor cells. This further supports the critical role of HMGB1 as a key Necroptosis.Sig modeling gene in the development of malignant tumors.

## Result

### Pan-cancer analysis of the association between necroptosis-related gene abundance and immune resistance

**Figure 1:**
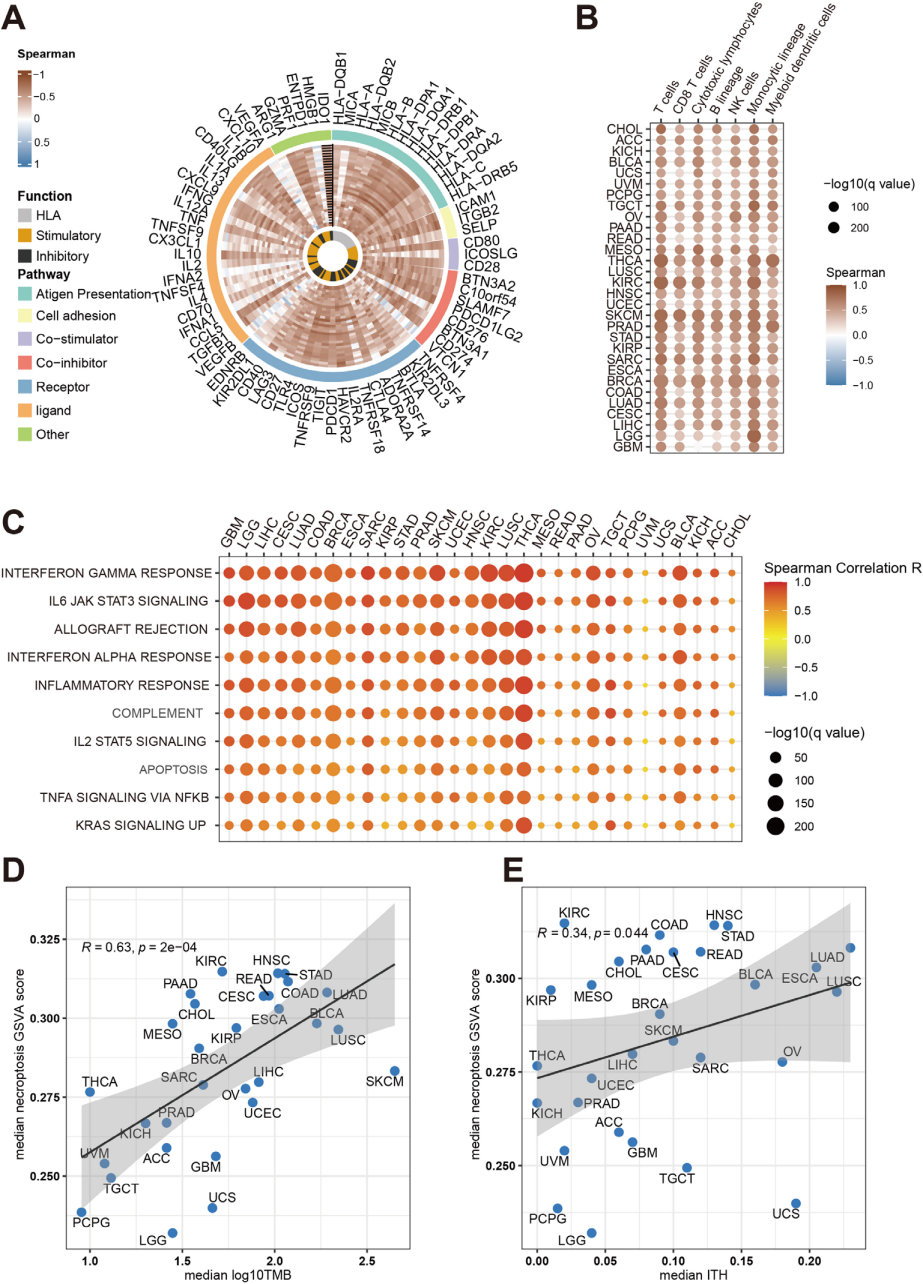
Comprehensive analysis of necroptosis associations with immune infiltration and tumor traits across cancers in the TCGA cohort. **(A)** The Circos plot illustrates the relationship between necroptosis pathway activity (measured by GSVA scores) and immune gene expression across multiple cancer types. The color gradient reflects Spearman correlation values, ranging from -1 to 1. Gene functions are categorized as antigen presentation, HLA, stimulatory, inhibitory, and other immune-related functions. **(B)** This heatmap shows how necroptosis pathway activity correlates with immune cell infiltration (e.g., T cells, B cells, macrophages) in different cancers. Dot size indicates statistical significance (-log10(q value)), while color represents the Spearman correlation coefficient. **(C)** A bubble heatmap demonstrates the association between necroptosis pathway activity and key immune-related pathways, such as interferon gamma response, IL6 JAK STAT3 signaling, and inflammatory response, across cancer types. Dot size reflects statistical significance (-log10(q value)), and color represents the correlation strength (Spearman R). **(D)** Scatter plot depicting the correlation between median necroptosis pathway activity scores and median tumor mutational burden (TMB) across cancer types. Each point corresponds to a different cancer type, with the shaded area representing the confidence interval of the regression line. **(E)** Scatter plot showing the association between median necroptosis pathway activity scores and median intratumor heterogeneity (ITH) for various cancers. Similar to panel D, the Spearman correlation coefficient (R) and p-values are indicated in both plots.

In this study, we investigated the association between necroptosis-related gene abundance and immune resistance, aiming to uncover novel insights into their role in cancer immunotherapy. Using GSVA, we computed necroptosis scores across the TCGA pan-cancer cohort, revealing a significant positive correlation between necroptosis scores and immune-related gene expression across 30 distinct cancer types (**Figure 1A**). These findings suggest a broad impact of necroptosis on immune modulation within the tumor microenvironment (TME).To further understand this impact, we examined immune cell infiltration in tumors with high necroptosis scores, observing a notable increase in immune cell presence, underscoring a link between necroptosis and immune activation. Importantly, our analysis also revealed positive correlations between necroptosis scores and both intratumor heterogeneity (ITH) and tumor mutational burden (TMB) (**Figures 1D, E**), suggesting that necroptosis influences anti-tumor immune responses by modulating immune cell activity and regulating tumor heterogeneity. These results provide new evidence that tumors with elevated necroptosis scores display enhanced anti-tumor immune responses, positioning necroptosis as a potential predictive biomarker for cancer immunotherapy efficacy. Our findings thus contribute to the understanding of necroptosis as a mechanism influencing immune resistance, with implications for identifying patients who may benefit most from immunotherapeutic interventions.

### Predicting immunotherapy outcomes using necroptosis gene signature

**Figure 2:**
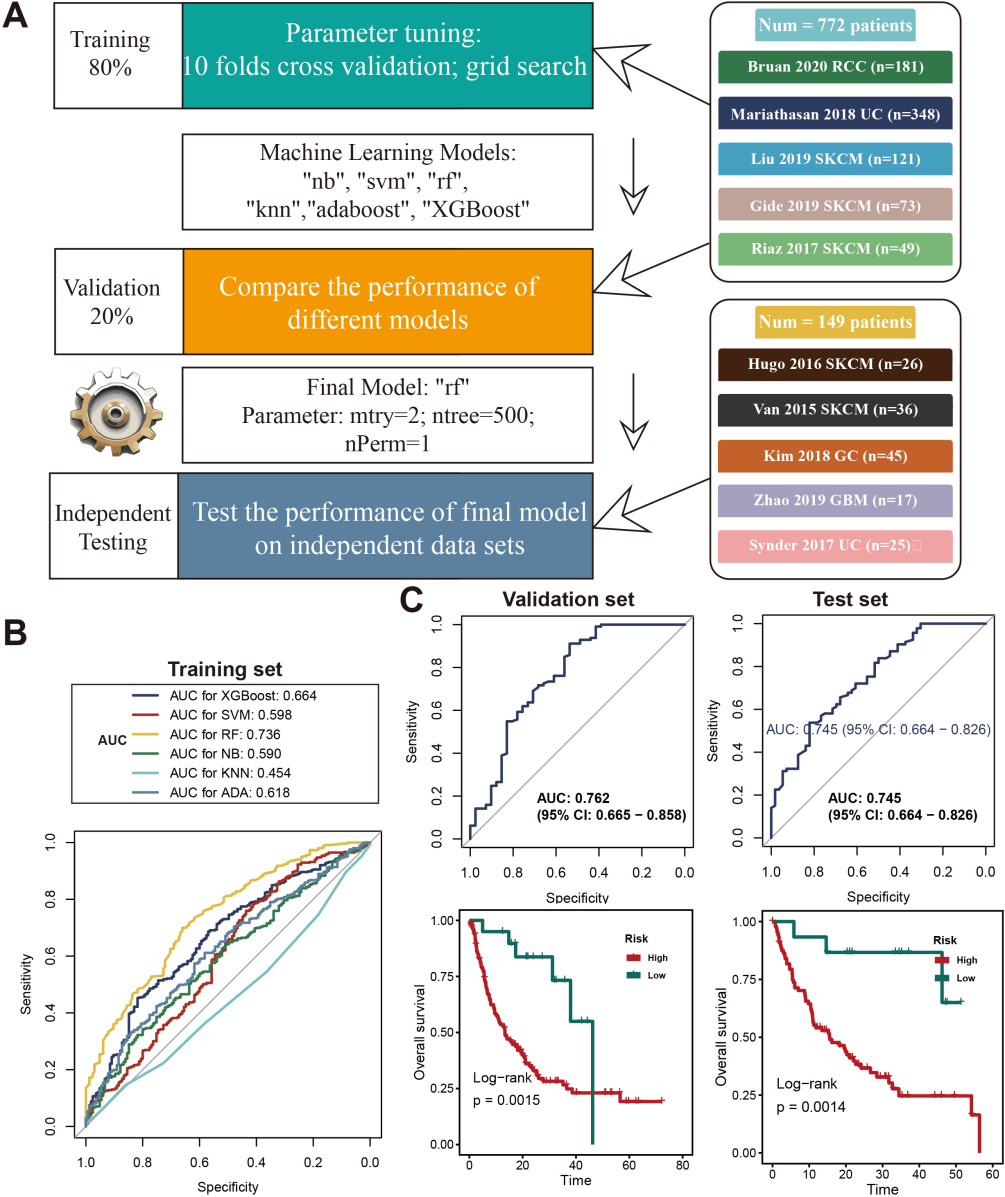
Development and evaluation of a machine learning model for predicting the necroptosis signature. **(A)**Diagram outlining the machine learning process used to create and validate the necroptosis signature model. The data was divided into 80% for training and 20% for validation. Parameter tuning was done using 10-fold cross-validation and grid search across various models (e.g., “nb”, “svm”, “rf”, “knn”, “adaboost”, “XGBoost”). The final model selected was Random Forest (rf), with optimized parameters, and was tested on independent datasets from studies like Braun 2020 RCC and Kim 2018 GC. **(B)** ROC curves displaying the performance of different machine learning models on the training set, with AUC values for models such as XGBoost, SVM, RF, KNN, and AdaBoost. Random Forest achieved the highest AUC of 0.736. **(C)** ROC curves showing the final Random Forest model’s performance in the validation set (AUC = 0.762, 95% CI: 0.665–0.858) and test set (AUC = 0.745, 95% CI: 0.664–0.826). Kaplan-Meier survival curves indicate differences in overall survival (OS) between high-risk and low-risk groups in both validation and test sets, with significant p-values from log-rank tests (p = 0.0015 and p = 0.0014, respectively).

Acknowledging the critical role of necroptosis in orchestrating anti-tumor immune responses, we developed a predictive model based on necroptosis marker genes to optimize immunotherapy strategies. RNA-Seq data and clinical records from ten immune checkpoint inhibitor (ICI) cohorts were collected. Using the ABESS algorithm, five key necroptosis marker genes (ANKRD28, CREB3L2, ISG20, SLAMF7, MEI1) were identified. The workflow for model development is depicted in **Figure 2A**, where six different machine learning algorithms were employed, utilizing 10-fold cross-validation and grid search for parameter optimization to generate prediction models. Among these models, the Random Forest (RF) model exhibited the best performance, with an AUC of 0.713 (**Figure 2B**). Subsequently, the model was validated on the validation and independent testing sets, yielding AUCs of 0.71 and 0.74, respectively (**Figure 2C**). To further assess the model’s predictive capacity for overall survival (OS), ICI-treated patients were stratified into high-risk and low-risk groups. Kaplan-Meier survival analysis demonstrated that the low-risk group exhibited significantly prolonged OS in both the validation and testing sets (P < 0.01) (**Figure 2C**, right).

### Comparison of necroptosis gene signature with other predictive gene signatures

**Figure 3:**
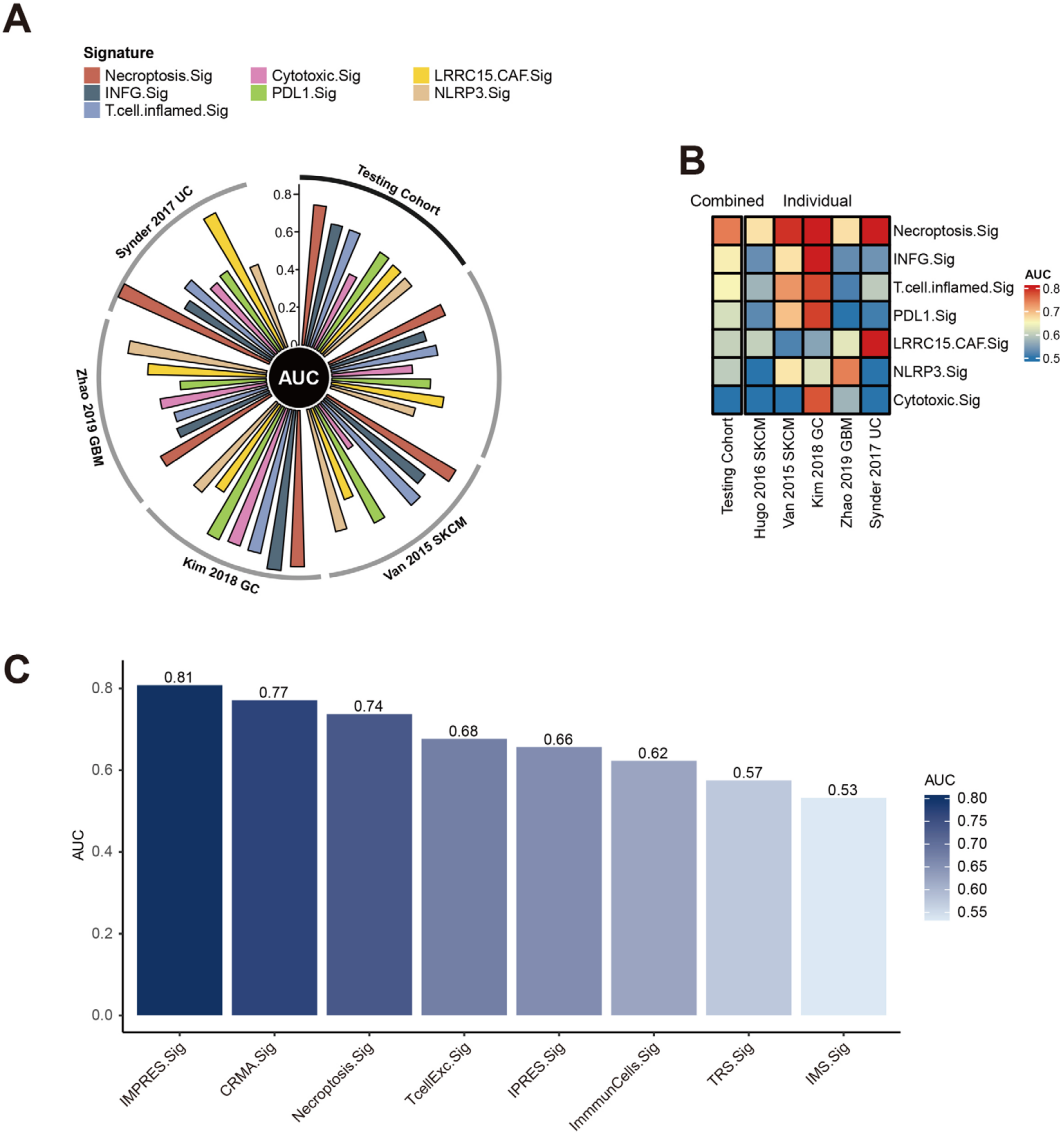
Performance of necroptosis signature across cancer cohorts. **(A)** Circos plot displaying the Area Under the Curve (AUC) values for multiple immune-related signatures, including Necroptosis.Sig and Cytotoxic.Sig, across various testing cohorts such as Snyder 2017 UC, Van 2015 SKCM, Kim 2018 GC, and Zhao 2019 GBM. The radial arrangement highlights the predictive performance of each signature in different cohorts. **(B)** Heatmap showing the AUC values of combined and individual necroptosis-related signatures across different datasets. The color scale ranges from 0.5 to 0.8, with warmer tones indicating higher predictive accuracy. **(C)** Bar plot comparing AUC values for various immune and necroptosis-related signatures, such as IMPRES.Sig, CRMA.Sig, and Necroptosis.Sig, highlighting their predictive strength. Higher bars reflect better performance.

We evaluated the predictive power of the necroptosis gene signature (Necroptosis.Sig) against other immune checkpoint inhibitor (ICI)-related gene signatures. In a direct comparison with a spectrum of pan-cancer signatures, including INFG.Sig (26), T.cell.inflamed.Sig (26), PDL1.Sig (27), LRRC15.CAF.Sig (28), NLRP3.Sig (29), and Cytotoxic.Sig (30), Necroptosis.Sig emerged as the most effective predictor in the testing set, achieving an AUC of 0.74, with INFG.Sig trailing closely behind at an AUC of 0.66 (**Figure 3A**). Necroptosis.Sig demonstrated superior performance across all evaluated cohorts, spanning three distinct cancer types: melanoma (SKCM), glioblastoma (GBM), and gastric cancer (GC), underscoring its versatility as a predictive model for ICI responsiveness across various malignancies (**Figure 3B**). When compared with signatures specifically tailored for melanoma (CRMA.Sig, IMPRES.Sig, IPRES.Sig, TcellExc.Sig, ImmunCells.Sig, IMS.Sig, and TRS.Sig), Necroptosis.Sig remained a leading predictor, achieving an AUC of 0.72 in predicting ICI response in melanoma patients. However, IMPRES.Sig and CRMA.Sig outperformed Necroptosis.Sig in this subset, registering slightly higher AUCs of 0.81 and 0.77, respectively (**Figure 3C**).

### Functional analysis of necroptosis gene signature in tumor immune microenvironment

**Figure 4:**
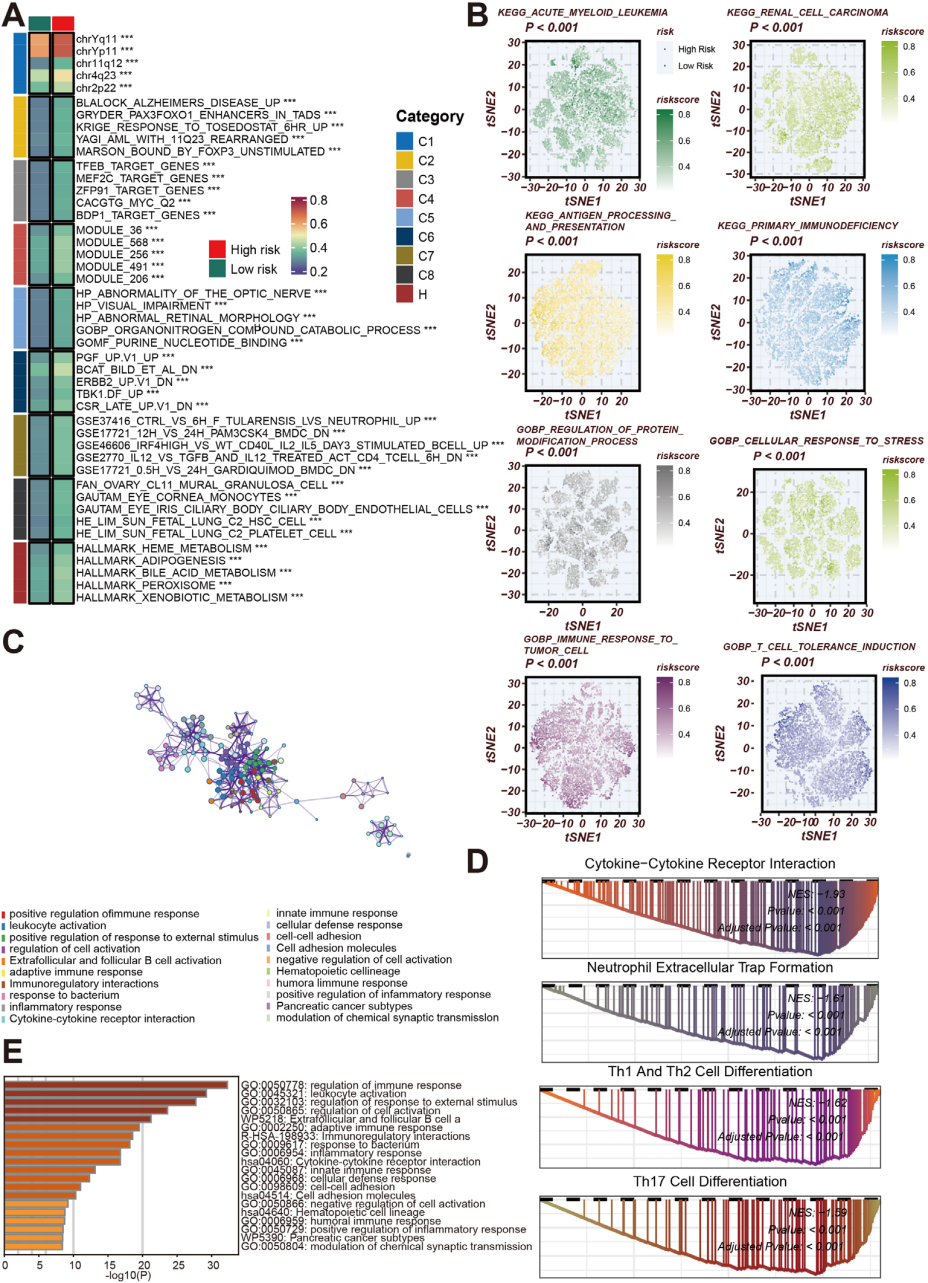
Functional and Pathway Analysis of Necroptosis Gene Signature in Cancer. **(A)** Heatmap illustrating associations between gene sets and necroptosis risk across various cancer cohorts. Gene modules are classified into functional groups (C1 to C8), with risk categories annotated as high or low based on necroptosis risk scores. The gene sets highlighted include those related to cell cycle, metabolic processes, and immune regulation. **(B)** t-SNE plots showing the distribution of necroptosis risk scores in different cancer types, including acute myeloid leukemia, renal cell carcinoma, and antigen processing pathways. The risk scores are color-coded from low (green) to high (red), indicating the association between necroptosis and key biological pathways such as protein modification and stress response. **(C)** Network diagram depicting interactions between gene modules involved in cell cycle regulation and chromosome segregation. These gene modules highlight the complex interactions between pathways associated with tumor progression and necroptosis. **(D)** Bar graph showing the top enriched Gene Ontology (GO) terms related to immune response, cell activation, and inflammatory processes. The length of the bars corresponds to the significance level of enrichment (-log10(P)) for each GO term. **(E)** Gene Set Enrichment Analysis (GSEA) plots for key enriched pathways, including cytokine-cytokine receptor interaction, neutrophil extracellular trap formation, and Th1/Th2/Th17 cell differentiation. The plots show enrichment scores and p-values, emphasizing the involvement of necroptosis in immune cell activation and differentiation pathways.

Necroptosis, a unique form of programmed cell death distinct from classical apoptosis, is driven by the activation of key molecular pathways involving RIPK1, RIPK3, and MLKL. Unlike apoptosis, necroptosis causes cell membrane rupture, releasing various immune-stimulating factors that trigger an inflammatory response. This distinct role of necroptosis in the TME has drawn considerable attention in recent cancer research. To further elucidate the impact of the Necroptosis Gene Signature on tumor immune evasion and anti-tumor immune responses, we conducted an in-depth analysis. Our findings, visualized in a heatmap (**Figure 4A**), highlight the association between necroptosis-related gene sets and necroptosis risk across diverse cancer types. These insights underscore the potential of necroptosis as a regulatory mechanism within the TME, advancing our understanding of how necroptosis may influence immune resistance and support novel strategies for enhancing cancer immunotherapy.

The gene modules are classified into different functional groups (C1 to C8), and patients are divided into high-risk and low-risk groups based on necroptosis risk scores. Notably, these gene sets are primarily involved in key biological pathways such as cell cycle regulation, chromosome segregation, and metabolic processes, indicating a close relationship between necroptosis and cellular proliferation and metabolism. Next, t-SNE analysis (**Figure 4B**) further demonstrates the distribution of necroptosis risk scores across various cancer types, including acute myeloid leukemia, renal cell carcinoma, and antigen processing pathways. Necroptosis enhances the immune system’s ability to recognize tumor cells by promoting antigen presentation, thereby influencing anti-tumor immune responses. Through color coding (ranging from low to high risk), the relationship between necroptosis and key biological functions, such as protein modification, immune response, and cellular stress adaptation, is clearly displayed. Additionally, the network diagram (**Figure 4C**) highlights the complex interactions between gene modules involved in cell cycle control and chromosome segregation, suggesting that necroptosis not only affects cell death but may also regulate cancer progression by altering cellular proliferation and differentiation. At the same time, GO enrichment analysis (**Figure 4D**) reveals a strong association between necroptosis genes and immune responses, particularly in terms of leukocyte activation, cytokine signaling, and inflammation regulation. This further demonstrates the critical role of necroptosis in triggering anti-tumor immune activity. Finally, the GSEA bar graph (**Figure 4E**) shows the enrichment of necroptosis-related pathways, including cytokine-cytokine receptor interaction, neutrophil extracellular trap formation, and Th1/Th2/Th17 cell differentiation, all of which are immune-related processes. These results collectively emphasize the central role of necroptosis in immune cell activation, differentiation, and tumor immunity, further validating its potential as a therapeutic target in cancer immunotherapy.

### The relationship between necroptosis-related gene signatures and immune infiltration across various cancer types

**Figure 5:**
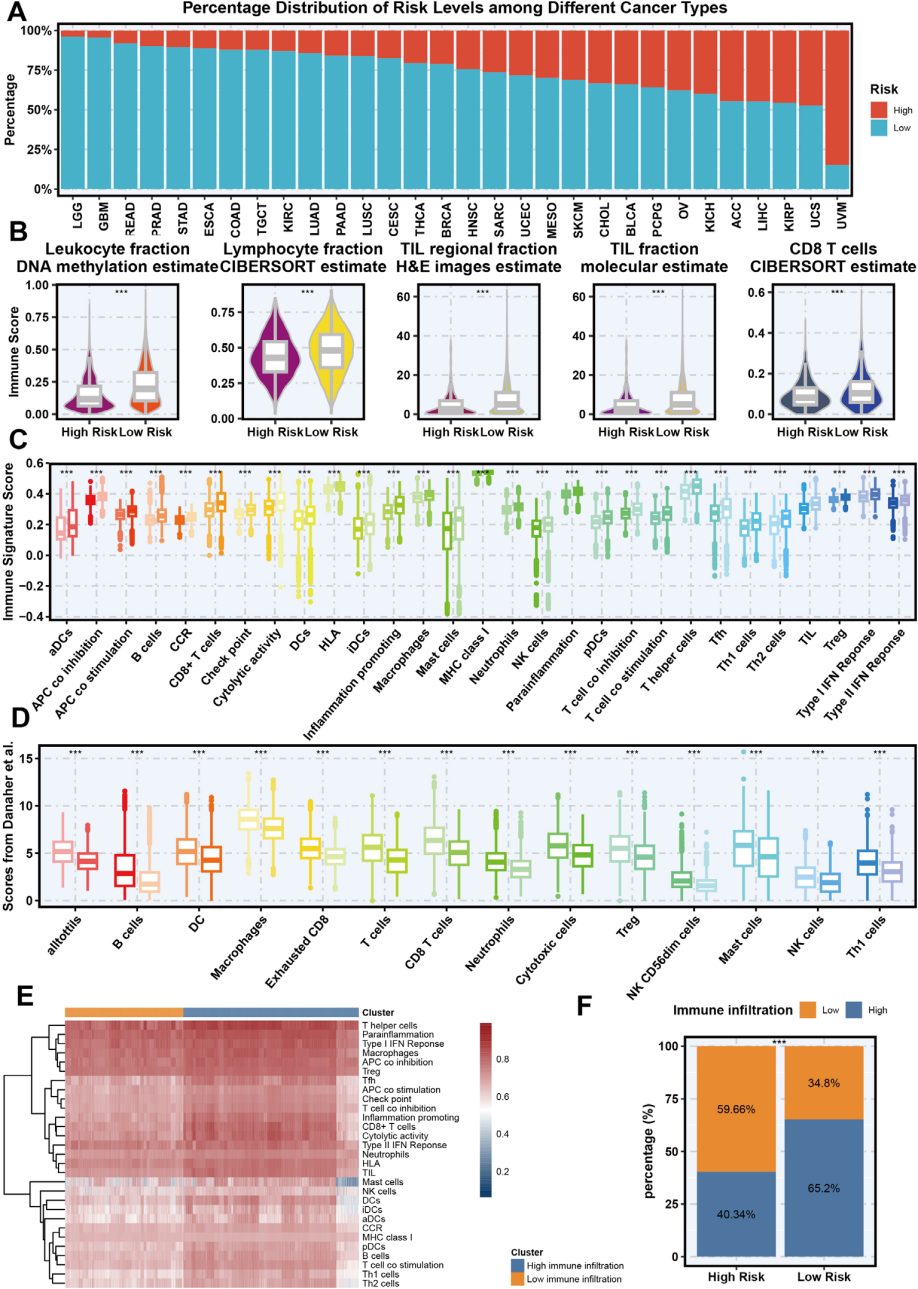
The association between necroptosis signature and immune infiltration across different cancer types. **(A)** Percentage distribution of risk levels (High vs Low) based on necroptosis signature across different cancer types from the TCGA cohort. **(B)** Violin plots showing the relationship between necroptosis signature (high and low risk) and different immune-related metrics, including leukocyte fraction (DNA methylation estimate), lymphocyte fraction (CIBERSORT estimate), tumor-infiltrating lymphocyte (TIL) regional fraction (H&E images estimate), TIL fraction (molecular estimate), and CD8 T cells (CIBERSORT estimate). **(C)** Boxplot of immune signature scores across various immune cell populations, comparing high-risk and low-risk groups based on the necroptosis signature. **(D)** Scores from Danaher et al. indicating the association between necroptosis signature and specific immune cell populations in high-risk and low-risk groups. **(E)** Heatmap showing the clustering of different immune cell types based on their association with necroptosis signature risk groups. **(F)** Bar plot representing the proportion of high and low immune infiltration in high-risk and low-risk necroptosis signature groups.

The relationship between necroptosis-related gene signatures and immune infiltration across various cancer types is analyzed to highlight novel findings and their implications in cancer immunotherapy. We explored how Necroptosis.Sig correlates with immune infiltration across diverse cancer types, aiming to understand its impact on the immune microenvironment. **Figure 5A** presents a bar graph illustrating the distribution of high-risk and low-risk groups based on Necroptosis.Sig across multiple cancer types. This analysis reveals that certain cancers, such as uveal melanoma and uterine carcinosarcoma, exhibit a higher proportion of high-risk patients, whereas others, like low-grade glioma, predominantly feature low-risk patients, suggesting necroptosis plays distinct roles across different cancers. In **Figure 5B**, violin plots compare immune-related metrics between high-risk and low-risk groups, showing significantly elevated scores in the low-risk group for leukocyte fraction, lymphocyte fraction, tumor-infiltrating lymphocyte (TIL) fractions, and CD8+ T cell infiltration. These findings indicate a more active immune system in the low-risk group. **Figure 5C** expands on this with boxplots showing that high-risk groups display markedly lower immune signature scores across multiple immune cell types, particularly antigen-presenting cells (APCs), B cells, and CD8+ T cells, implying a weaker immune response in the high-risk group. Further validation through Danaher et al.’s scoring analysis in **Figure 5D** reinforces these results, as the low-risk group shows significantly higher immune activity in cell populations such as B cells, dendritic cells, macrophages, and CD8+ T cells, emphasizing a robust immune presence. Additionally, the heatmap in **Figure 5E** clusters immune cells by risk group, revealing strong associations between the low-risk group and immune cell activity, particularly in Th1 cells, dendritic cells, and NK cells, thus supporting the view that the low-risk group has a more active immune microenvironment. Finally, **Figure 5F** summarizes immune infiltration differences between the two groups, illustrating that 59.66% of high-risk patients have low immune infiltration, while 65.2% of low-risk patients demonstrate high immune infiltration. This comprehensive analysis highlights that Necroptosis.Sig is inversely related to immune infiltration, with higher necroptosis risk correlating with reduced immune presence and potentially influencing tumor immune evasion and patient prognosis. This reorganization underscores our key finding that Necroptosis.Sig’s relationship with immune infiltration varies by cancer type and risk group, contributing novel insights to the field of cancer immunotherapy.

### Necroptosis.Sig influence various genomic and immunological features of pan-cancer patient

**Figure 6:**
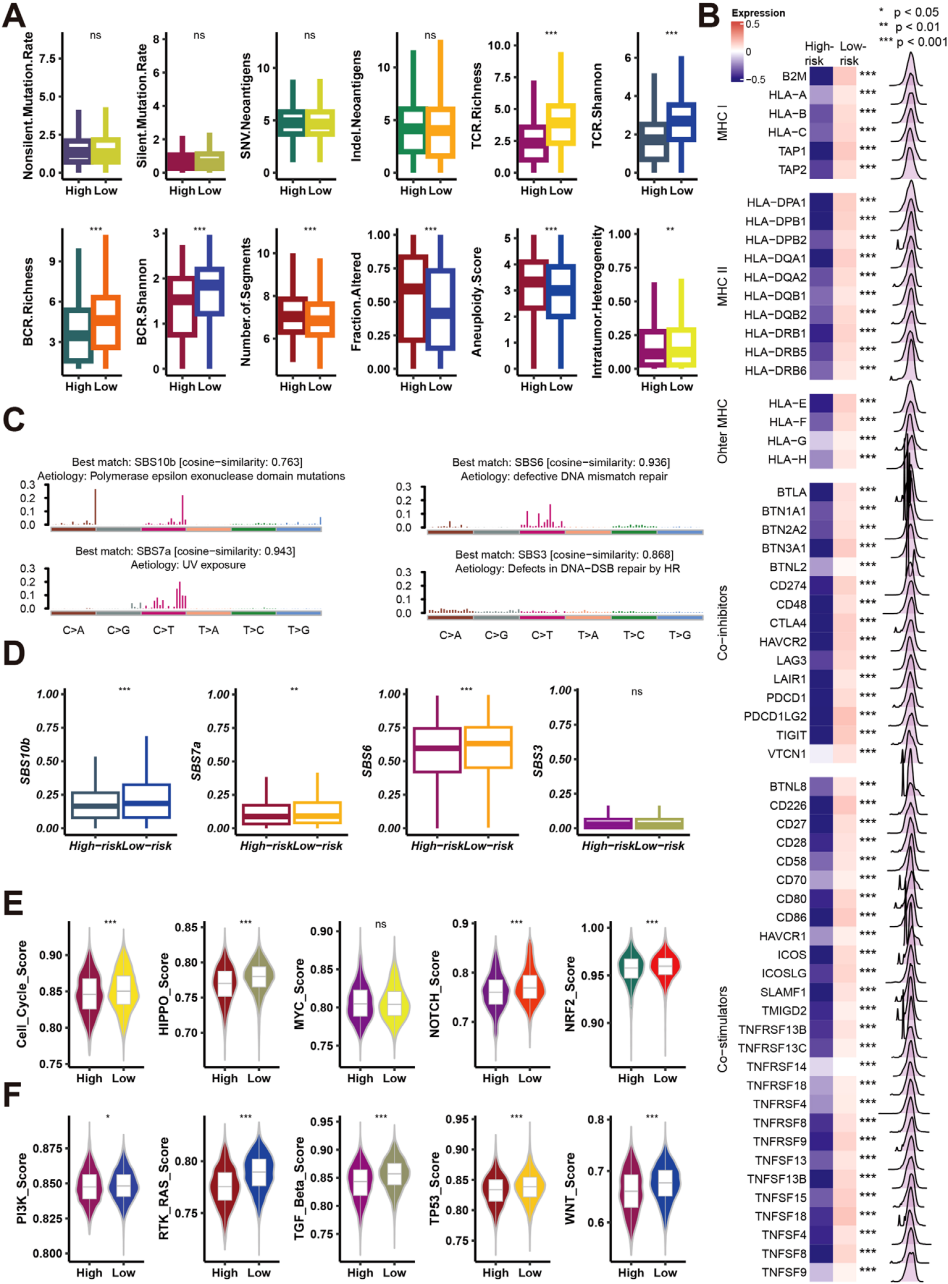
Analysis of mutation rates, immune signatures, and pathway activities in high- and low-risk groups. **(A)** Box plots comparing mutation rates, immune receptor richness (BCR, TCR), and tumor-related features (e.g., aneuploidy score, intratumor heterogeneity) between high- and low-risk groups. Significance is indicated as *p < 0.05, **p < 0.01, ***p < 0.001, ns = not significant. **(B)** Heatmap showing expression differences in MHC class I/II genes, other MHC molecules, and immune checkpoint inhibitors between high- and low-risk groups, with p-values denoting significance. **(C)** Mutation signature plots showing cosine similarity for various mutation signatures (SBS10b, SBS6, SBS7a, SBS3) between risk groups, linked to different etiologies such as UV exposure and DNA repair defects. **(D)** Box plots comparing the contribution of mutation signatures (SBS10b, SBS7a, SBS6, SBS3) between high- and low-risk groups. **(E)** Violin plots showing pathway activity scores (e.g., cell cycle, HIPPO, MYC, NOTCH, NRF2) between high- and low-risk groups. **(F)** Violin plots comparing additional pathway activities (e.g., PI3K, RTK-RAS, TGF-beta, TP53, WNT) between high- and low-risk groups. Significance is indicated as in **(A)**.

Next, this study delves into the relationship between high-risk and low-risk groups, as defined by Necroptosis.Sig, in terms of genomic and immunological characteristics. **Figure 6A** compares the mutation rate, neoantigen burden, T-cell receptor (TCR) and B-cell receptor (BCR) diversity, aneuploidy score, and intratumor heterogeneity between the two groups. The results show that the low-risk group has higher TCR and BCR diversity, indicating a more diverse immune repertoire, while the high-risk group exhibits greater genomic instability, with higher aneuploidy scores and intratumor heterogeneity. **Figure 6B** presents a heatmap of the differential expression of MHC-I and MHC-II molecules, as well as immune checkpoint inhibitors, with the low-risk group showing higher MHC expression, and the high-risk group displaying increased immune checkpoint expression, suggesting potential immune evasion mechanisms in the high-risk cohort. **Figure 6C** illustrates mutation signatures related to DNA repair defects, which are more pronounced in the high-risk group. **Figure 6D** confirms these findings by showing that mutation signatures associated with polymerase epsilon mutations, UV exposure, DNA mismatch repair defects, and homologous recombination defects are significantly higher in the high-risk group. **Figures 6E, F** compare the activity of several oncogenic pathways, such as Cell Cycle, MYC, HIPPO, TGF-beta, and WNT, with the high-risk group showing higher activity in these pathways, suggesting more aggressive tumor behavior and potential therapeutic vulnerabilities. Overall, this figure indicates that high-risk patients with elevated Necroptosis.Sig exhibit greater genomic instability, more active oncogenic pathways, and increased expression of immune checkpoint molecules, contributing to a more aggressive and immunosuppressive tumor microenvironment.

### HMGB1 knockdown suppresses A549 lung cancer cell proliferation and tumor growth

**Figure 7:**
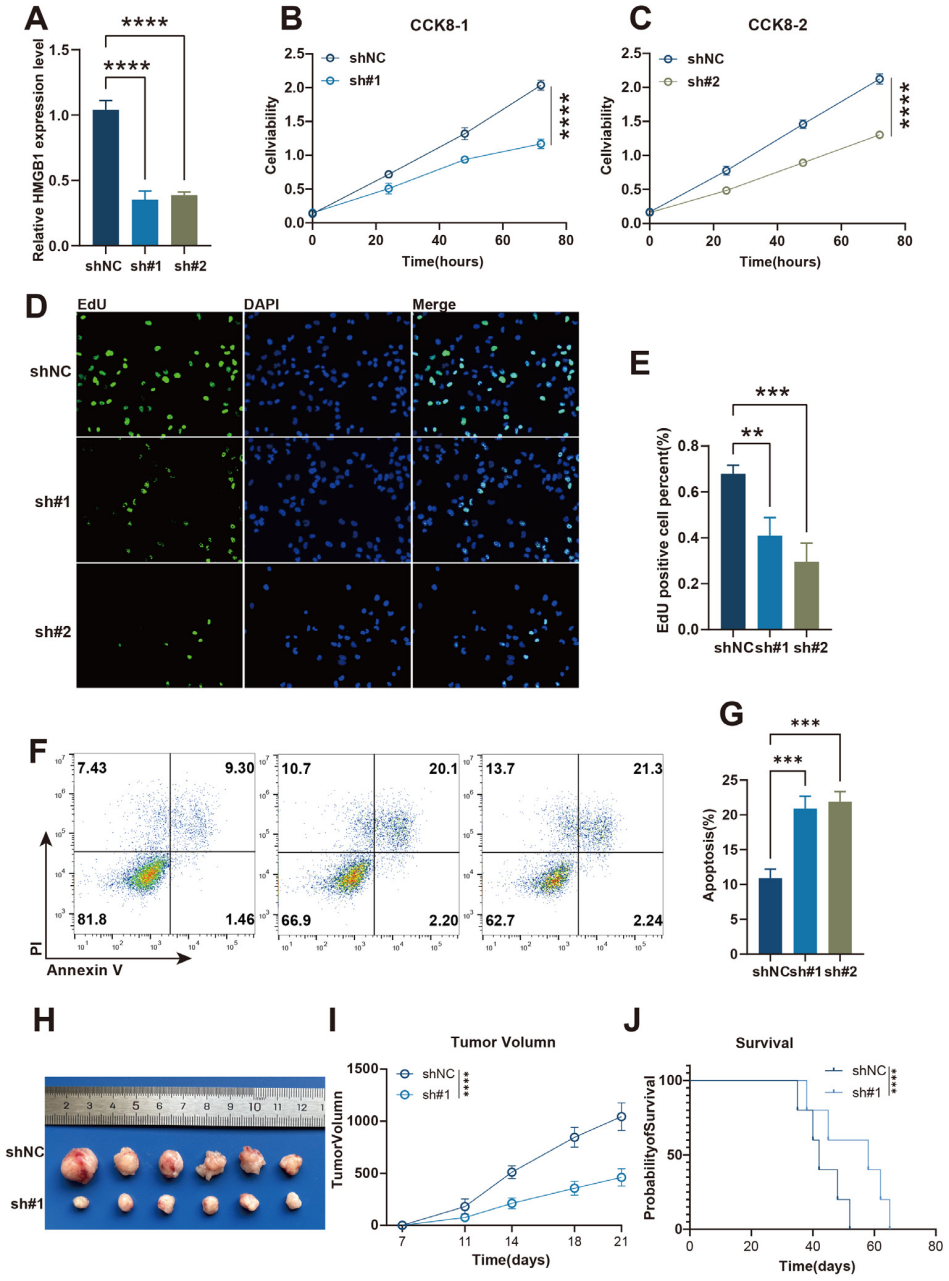
The knockdown of HMGB1 inhibits A549 lung cancer cell proliferation and promotes apoptosis *in vitro* and *in vivo*. **(A)** Relative expression levels of HMGB1 in shNC, sh#1, and sh#2 A549 lung cancer cells, as measured by qRT-PCR. **(B, C)** Cell viability analysis using CCK-8 assay in shNC and sh#1 or sh#2 A549 lung cancer cells at different time points. **(D)** EdU incorporation assay to measure cell proliferation in shNC, sh#1, and sh#2 A549 lung cancer cells. **(E)** Quantification of EdU-positive A549 lung cancer cells in shNC, sh#1, and sh#2 groups. **(F)** Flow cytometry analysis of apoptosis in shNC, sh#1, and sh#2 A549 lung cancer cells. **(G)** Quantification of apoptosis rates in shNC, sh#1, and sh#2 A549 lung cancer groups. **(H)** Tumor images from xenograft models showing the tumor sizes from shNC and sh#1 groups. **(I)** Tumor volume measurements over time in shNC and sh#1 groups. **(J)** Kaplan-Meier survival curves comparing the survival of mice in the shNC and sh#1 groups. Statistical significance: p < 0.05, p < 0.01, p < 0.001, ****p < 0.0001.

To further investigate the role of HMGB1, one of the modeling genes of Necroptosis.Sig, in A549 lung cancer cells and animal experiments, we conducted a series of analyses. The results showed that knocking down HMGB1 significantly reduced its relative expression levels (**Figure 7A**) and inhibited cell proliferation. In the CCK-8 assay, cells with HMGB1 knockdown exhibited a significantly slower proliferation rate at different time points (**Figures 7B, C**). The EdU staining assay further demonstrated that the percentage of EdU-positive cells was significantly reduced in the HMGB1 knockdown group (**Figures 7D, E**), confirming the suppression of cell proliferation. Meanwhile, flow cytometry analysis indicated that the apoptosis rate was significantly increased in HMGB1 knockdown cells (**Figures 7F, G**). In the mouse xenograft model, tumors in the HMGB1 knockdown group were noticeably smaller, and tumor growth was significantly slower (**Figures 7H, I**). Furthermore, Kaplan-Meier survival curves showed that mice in the HMGB1 knockdown group had significantly improved survival rates (**Figure 7J**). Overall, these results suggest that HMGB1 plays a critical role in tumor cell proliferation, apoptosis, and tumor growth, further supporting its importance as a Necroptosis.Sig modeling gene in tumor development and immune evasion.

## Materials and methods

### Pan-cancer transcriptomic and ICI RNA-Seq data

We retrieved multi-omics datasets from the TCGA Pan-cancer collection via UCSC Xena (https://xenabrowser.net/datapages/) to explore the relationship between Necroptosis.Sig and immunosuppression across cancers. DLBC, LAML, and THYM were excluded due to their high immune cell content, which could introduce bias. The necroptosis gene sets were obtained from the GSEA database (https://www.gsea-msigdb.org/gsea/index.jsp). To address the potential batch effects arising from different tumor cohorts within the TCGA dataset, we applied the ComBat algorithm from the sva package (31), which effectively adjusts for batch effects while preserving biological variation across cancer types.

To validate the predictive capability of Necroptosis.Sig, we systematically collected transcriptomic data and clinical information from 10 pretreated ICI RNA-Seq cohorts. These cohorts include 5 melanoma (SKCM) cohorts (32–36), 2 urothelial carcinoma (UC) cohorts (37, 38), 1 glioblastoma multiforme (GBM) cohort (39), 1 gastric cancer (GC) cohort (40), and 1 renal cell carcinoma (RCC) cohort (41). Anti-PD-1, anti-PD-L1, anti-CTLA-4, and anti-PD-(L)1 + anti-CTLA-4 combination therapies were applied to 6, 2, 1, and 1 cohort, respectively. The Hugo 2016 cohort contains 27 pretreated tumor samples from 26 patients (32), while the Zhao 2019 cohort includes 34 pretreated tumor samples from 17 patients (39).

### Pan-cancer analysis of the association between different immune functions and tumor characteristics

Spearman correlation analysis was used to evaluate the relationship between immune checkpoint gene expression and different immune functions. GSVA (Gene Set Variation Analysis) (42) was employed to assess the activity of immune-related signaling pathways (e.g., interferon gamma response, IL6-JAK-STAT3 signaling) across various cancer types. Additionally, the relationship between the median necroptosis GSVA score and tumor mutation burden (TMB), as well as intratumor heterogeneity (ITH), was analyzed, and linear regression trend lines were plotted on scatter plots.

### ICI RNA-Seq cohorts

We compiled RNA-Seq and clinical data from 10 Immune Checkpoint Inhibitor (ICI) RNA-Seq datasets, covering five cutaneous melanoma datasets, two urothelial carcinoma datasets, one glioblastoma dataset, one gastric cancer dataset, and one renal cell carcinoma dataset. Different immunotherapies targeting PD-1, CTLA-4, and PD-L1 were administered across cohorts. To address batch effects, we used the ComBat-seq method.

### Clinical outcomes

The primary outcomes were Objective Response Rate (ORR) and Overall Survival (OS), with ORR evaluated using RECIST v1.1 for most cohorts, except one, which used *irRECIST* guidelines. Patients were categorized as responders (Complete/Partial Response) or non-responders (Stable/Progressive Disease).

### Development and validation of a predictive signature for the ICI dataset

We developed a predictive signature for the ICI dataset by creating a combined cohort of 772 samples from five ICI RNA-Seq datasets. This cohort was split into a training set (80%, n=618) and a validation set (20%, n=154), with the remaining datasets (n=149) used as an independent test set. After training various models, the top-performing one was selected from the training set and subsequently tested on both the validation and independent datasets to evaluate its predictive performance. The Necroptosis.Sig signature was then compared to six pan-cancer and seven melanoma-specific signatures for its accuracy in predicting ICI response within the test set. Additionally, melanoma-specific signatures were further assessed using two separate melanoma cohorts.

### Training and hyperparameter adjustment

Using the abess algorithm, we identified key necroptosis marker genes. Six machine learning models (SVM, Naive Bayes, Random Forest, KNN, AdaBoost, XGBoost) were built with 10-fold cross-validation and grid search for tuning (43).

### Functional and immune analysis

We performed gene set enrichment analysis (GSEA) and ssGSEA using MSigDB, GSVA, and clusterprofiler R packages to analyze functional and immune characteristics (44, 45). To assess immune infiltration, CIBERSORT was used to estimate the abundance of 22 immune cell subsets in the tumor microenvironment from normalized transcriptomic data (46). Tumor-infiltrating lymphocytes (TILs) were evaluated using both genomic and histopathological data, while lymphocyte fractions were estimated based on CIBERSORT results and DNA methylation profiles. Immune infiltration scores for 14 immune cell types were calculated using a 60-marker gene signature and validated through immunohistochemistry and flow cytometry. We also assessed 29 immune signatures, quantifying enrichment levels across individual samples with the ssGSEA method. Additionally, immunogenomic indicators such as intratumor heterogeneity (ITH), TCR, and BCR diversity were derived from previous studies, utilizing the ABSOLUTE algorithm for copy number aberrations and Shannon entropy for receptor diversity.

### Cell culture

Protocol for A549 lung cancer mouse cell culture: Begin by preparing necessary materials and sterilizing the clean bench. For cell revival, quickly thaw the cryogenic tube, mix it with preheated medium, centrifuge, and replace with fresh medium. Cultivate the cells in a CO_2_ incubator. When cell density reaches 80%-90%, digest with trypsin, centrifuge, resuspend the cells, and passage them at a 1:2 ratio, continuing cultivation in the CO_2_ incubator. For cryopreservation, wash and digest the cells, centrifuge, add cryopreservation solution, and gradually freeze before storage.

### ShRNA stable cell line construction

Using a transfection reagent, the constructed plasmid is introduced into the target cell line. The cells are then cultivated in medium containing puromycin to select for and eliminate non-transfected cells. The resulting cells, which stably express the specific shRNA, constitute the stable cell line. Lastly, these cells are expanded and characterized to confirm the gene silencing effect. The target sequences for HMGB1 were as follows: sh#1, CCGTTATGAAAGAGAAATGAA; and sh#2, CCCAGATGCTTCAGTCAACTT.

### Real-time quantitative PCR

Total RNA was successfully extracted using TRIzol reagent from Sigma-Aldrich. Following this, cDNA was synthesized utilizing the TOYOBO reverse transcription kit. In the qRT-PCR procedure, we employed SYBR Green reagent from Applied Biosystems and selected GAPDH as the internal reference gene. The relative expression of genes was calculated using the 2^-ΔΔCt^ method. All specific primers are listed in detail in the supplementary table, ensuring the reproducibility of the experiment. The primer sequences used are as follows: GAPDH: Forward Sequence 5’-GTCTCCTCTGACTTCAACAGCG-3’, Reverse Sequence 5’-ACCACCCTGTTGCTGTAGCCAA-3’. HMGB1: Forward Sequence 5’-GCGAAGAAACTGGGAGAGATGTG-3’, Reverse Sequence 5’-GCATCAGGCTTTCCTTTAGCTCG-3’.

### Flow cytometry for detection of cell apoptosis

Collect cell samples, perform appropriate treatment, stain with fluorescently labeled apoptosis detection reagents (Annexin V combined with PI), incubate in the dark for 25 minutes, and then analyze the fluorescent signals of the cell population using flow cytometry to distinguish between live cells, early apoptotic cells, late apoptotic/necrotic cells, thereby quantitatively assessing the level of apoptosis.

### Construction of a mouse subcutaneous tumor model

The 6-8 weeks-old female Balb/c-nu mice were purchased from Beijing Vital River Laboratory Animal Technology Co., Ltd. After digestion, the cells are resuspended in sterile saline and adjusted to an appropriate concentration. Subsequently, subcutaneous injections are administered at the lateral side of the mouse’s back at a dose of 5x10^5^ cells per mouse, ensuring the cells are accurately injected into the subcutaneous tissue. Following injection, the mice are observed regularly for their growth status and tumor development, with attention also paid to their daily care. Upon reaching the observation endpoint, the mice are euthanized by cervical dislocation.

### Statistical analysis

Data analysis was conducted in R 4.3.1, using the Wilcoxon rank-sum test for continuous variables and Spearman correlation for relationships. ROC curves and Kaplan-Meier survival analyses were performed using the pROC and survival packages, with p < 0.05 considered statistically significant.

## Conclusion

In this study, we constructed a necroptosis-related Sig that offers tremendous potential for personalized treatment in patients with pan-cancer. This necroptosis-related Sig represents a significant advancement in the field of oncology, as it offers a novel perspective on cancer biology and paves the way for innovative strategies in clinical decision-making and patient management. Furthermore, our research has deepened the understanding of the molecular mechanisms related to the tumor microenvironment (TME) influenced by the necroptosis-related Sig through a multi-omics approach.

### Ethical statement

All mice were kept in a specific pathogen-free environment and supplied with sterilized drinking water. All animal procedures were conducted in accordance with the ARRIVE guidelines and were approved by the Animal Ethics Committee of the Affiliated Huai’an Hospital of Xuzhou Medical University.

## Discussion

Necrotic apoptosis, a form of programmed cell death, contrasts with cytotoxic T cells that must directly interact with tumor cells (47, 48). Necrotic apoptosis represents a cellular death pathway distinct from apoptosis, often occurring when cells are subjected to extreme stress, leading to cell rupture and the release of cellular contents, which can promote inflammation and immune response (49, 50). Existing research suggests necrotic apoptosis can help eliminate tumor cells by attracting immune cells, such as macrophages and neutrophils, to the tumor microenvironment (51, 52). Moreover, interactions between necrotic cells and other immune components are pivotal in tumor immunity. Research indicates that the balance between necrotic cell death and apoptosis influences cancer progression and immune system activation (53–55). Studies have shown that the immune system’s response to necrotic cell death can either enhance or suppress tumor growth depending on the tumor’s microenvironment and the types of immune cells involved (56–58). Our study extends current understanding by exploring necrotic apoptosis’s potential role in enhancing immune checkpoint inhibitor (ICI) therapy efficacy across multiple cancer types. This investigation represents the first comprehensive analysis of necroptosis-related signatures in the context of pan-cancer ICI response (59). Additionally, our research sought to deepen understanding of the molecular mechanisms underpinning necrotic apoptosis through a multi-omics approach (60). This approach integrates transcriptomic, proteomic, and metabolomic data to reveal novel insights into the pathways that regulate necrotic cell death and its interplay with tumor immunity.

To the best of our knowledge, this is the first study to explore the relationship between necrotic apoptosis and the outcomes of ICIs therapy in pan-cancer (61). We developed a novel necrotic apoptosis signature, NecroticApop.Sig, through an integrative RNA-Seq analysis across multiple cancer types. This signature serves as a predictive biomarker for ICI response, addressing a critical need for robust predictors in immunotherapy. Our pan-cancer approach, utilizing TCGA and multiple ICI transcriptional cohorts, provides broad validation and demonstrates NecroticApop.Sig’s superior predictive performance, with an AUC exceeding 0.7 in multiple testing sets. This finding suggests that NecroticApop.Sig could enhance ICI treatment strategies by stratifying patients with higher accuracy than previously established biomarkers.

A key innovation in this study is the investigation of the immune landscapes within the tumor microenvironment. Our multi-omics analysis in the TCGA highlights significant immune cell infiltration differences between high- and low-risk groups, suggesting that NecroticApop.Sig not only predicts ICI response but also provides insight into tumor-immune dynamics. Specifically, low-risk groups exhibited greater immune cell activity, including higher CD8+ T cell and immunostimulatory cell levels, supporting NecroticApop.Sig’s predictive robustness across multiple cancers and emphasizing its value in tailoring immunotherapy strategies.

This research utilized six machine learning models to establish a stable and reliable signature, known as NecroticApop.Sig. NecroticApop.Sig was an innovative biomarker proficient in predicting responses to ICIs and effectively stratifying patients likely to experience survival benefits. Additionally, comparisons of NecroticApop.Sig with leading-edge signatures, including six pan-cancer and seven melanoma-specific markers, were conducted. NecroticApop.Sig demonstrated superior generalization capabilities over pan-cancer signatures and maintained robust performance across diverse cohorts.

While NecroticApop.Sig demonstrated promising results across 30 cancer types, comprehensive validation in a pan-cancer setting will require prospective clinical trials involving ICIs. Additionally, limited clinical annotations in certain RNA-Seq datasets, such as sex, age, tumor stage, tumor mutational burden (TMB), and intratumoral heterogeneity (ITH), restricts our ability to perform in-depth multivariate regression analyses. Addressing these limitations in future studies could enhance the biomarker’s predictive robustness.

Finally, while NecroticApop.Sig offers a powerful predictive tool, the exact roles of specific genes within the signature in regulating necroptosis remain unclear. Additional *in vitro* and *in vivo* studies are necessary to define these genes’ functions in necrotic cell death modulation and their potential implications for tumor immunity, further advancing the field of cancer immunotherapy.

### Outlook and limitations

This study highlights the potential role of necroptosis in predicting immune checkpoint inhibitor (ICI) responses, opening new avenues for further research. Future directions will involve comprehensive *in vitro* and *in vivo* experiments to validate the regulatory roles of NecroticApop.Sig genes in necroptosis and tumor immunity, particularly within diverse tumor microenvironments. However, several limitations warrant consideration. Firstly, our findings are primarily based on bioinformatics analyses, lacking in-depth experimental validation, which may limit the biological insights derived from the study. Extensive experimental research is necessary to confirm the accuracy and applicability of NecroticApop.Sig in different tumor contexts. Secondly, although NecroticApop.Sig shows promising predictive accuracy, it has not yet been validated with independent cohorts, and current findings may be constrained by sample representativeness and dataset diversity. To ensure the model’s robustness and broader applicability, larger and more diverse independent datasets are needed for further validation. Additionally, due to the complexity of tumor immunity and necroptosis, the clinical operability and predictive power of NecroticApop.Sig may be limited by individual patient variations and tumor heterogeneity. To enhance its clinical applicability and generalizability, we plan to collect broader independent datasets, conduct prospective studies, and perform refined analyses across various tumor types and patient subgroups. These efforts will contribute to strengthening the practical utility of NecroticApop.Sig in clinical settings.

## Data Availability

The original contributions presented in the study are included in the article/supplementary files, further inquiries can be directed to the corresponding author/s.
